# Evolution of the internal load and physical condition of wheelchair basketball players during the competitive season

**DOI:** 10.3389/fphys.2023.1106584

**Published:** 2023-03-15

**Authors:** Ander Romarate, Javier Yanci, Aitor Iturricastillo

**Affiliations:** ^1^ Faculty of Education and Sport, University of the Basque Country (UPV/EHU), Vitoria-Gasteiz, Spain; ^2^ Society, Sports and Physical Exercise Research Group (GIKAFIT), Physical Education and Sport Department, Faculty of Education and Sport, University of the Basque Country, UPV/EHU, Vitoria-Gasteiz, Spain

**Keywords:** disability, perceived load, fitness, training, wheelchair basketball, physical condition

## Abstract

The objectives of this study were to describe differentiated perceived training and match load (dRPE-L) of wheelchair basketball (WB) players during the whole season, to analyze the evolution of players’ physical condition changes during a full season and to analyze the association between dRPE-L and changes in physical condition during a full season. Nineteen Spanish Second Division WB players participated in this study. For a full season (10 months, 26 weeks), dRPE-L was assessed with the session-RPE method, separating respiratory (RPEres-L) and muscular (RPEmus-L) perceived load. The physical condition of the players was also assessed at four different times during the season (T1, T2, T3 and T4). The results showed a significantly higher total and average accumulated muscular RPE load (RPEmusTOT-L and RPEmusAVG-L) than total and average respiratory load (RPEresTOT-L and RPEresAVG-L) (*p* < 0.01; ES = 0.52–0.55). No significant changes were observed in the physical condition of the players at the different moments of the season. Moreover, a significant association was observed only between RPEresTOT-L and Repeated Sprint Ability standard deviation of 3 m (RSAsdec3m) (r = 0.90, *p* < 0.05). The results suggest that the competitive season represented considerable neuromuscular involvement in these players.

## 1 Introduction

Wheelchair basketball (WB) is one of the most popular Paralympic sports (Croft et al., 2010; Granados et al., 2015; Iturricastillo et al., 2015). It is practiced in more than 100 different countries, with more than 30,000 participants worldwide (Yanci et al., 2015). This sport involves athletes with different physical disabilities (e.g., spinal cord injury, amputees, spina bifida, joint and muscle limitations, *etc.*), which affect their ability to run, jump and pivot at speed and with the control, safety and endurance of able-bodied basketball players (Cavedon et al., 2015). In WB, five players compete against five players on a basketball court with the same dimensions but slight modifications to the rules of able-bodied basketball (IWBF, 2021). The International Wheelchair Basketball Federation (IWBF) designed a classification system based on 1) eligible impairments: impaired muscle power, impaired passive range of movement, limb deficiency, leg length difference, hypertonia, ataxia or athetosis; and 2) the minimum impairment criteria (MIC) for their eligible impairment (IWBF Player Classification Commision, 2021). After this classification stage, players are grouped into categories from 1.0 to 4.5 based on the players’ functional capacity to complete the skills necessary to play: pushing, pivoting, shooting, rebounding, dribbling, passing and catching (IWBF Player Classification Commision, 2021).

Like other team sports (Datson et al., 2014; Tavares et al., 2018), WB is a physically and physiologically demanding sport, where players must train and compete over long periods of time of up to 10–11 months, according to the competition calendar of national leagues and international events. During WB season, players support a great physical and physiological load (Yanci et al., 2014; Iturricastillo et al., 2017, 2018; Marszałek et al., 2019a) as they must respond to the demands of the training sessions and matches. Although some previous studies in WB have quantified the internal load during official matches (Iturricastillo et al., 2016) and training sessions (Yanci et al., 2014; Iturricastillo et al., 2017) or even combining match and training load (Schmid et al., 1998; Iturricastillo et al., 2016), it is still unknown how the physiological load of WB players evolves during a full season including regular competitions and play-offs (Iturricastillo et al., 2018). Knowing how the physiological load evolves throughout the season could be important for coaches and technical staff with the aim of maintaining/adjusting and/or modifying training loads to try to achieve the desired effects in improving players’ performance.

As well as quantifying match or training physiological load, multiple field tests have been used in order to determine WB players’ physical condition, such as, speed, acceleration, change of direction ability (CODA), strength and endurance (Yanci et al., 2015; Marszałek et al., 2019b; Iturricastillo et al., 2021). Usually, these tests have been measured in a simple way in order to determine the physical or physiological condition of the players (Goosey-Tolfrey, 2005; Chapman et al., 2010; Soylu et al., 2020) or to analyze the effects of a specific training program (Romarate et al., 2021). Even though the evolution of the physical condition throughout the season has been extensively analyzed in several able-bodied sports (Castillo et al., 2018; Mancha-Triguero et al., 2020), in WB few studies have analyzed the evolution of players’ physical condition longitudinally using different tests at different times in the season (Ayan et al., 2014; Iturricastillo et al., 2015). Although both Iturricastillo et al. (2015) and Ayan et al. (2014) have analyzed fitness changes in WB players, these studies only include the regular season (testing 3 and 2 times in the season, respectively), it is still unknown how WB players’ physical condition varies at other relevant times of the season (e.g., in play-offs). Thus, while in conventional team sports (Los Arcos et al., 2015; Djaoui et al., 2017; Flatt and Howells, 2019) and also in particular in basketball (Ferioli et al., 2018; Freitas et al., 2019; Sansone et al., 2021) the influence of the load on the development of players’ physical condition has been described, to the best of our knowledge only one study has analyzed this issue in WB and only during a short period (Iturricastillo et al., 2022). In this study, the evolution of the physical condition and the training internal load recording was only analyzed during a specific period of the season (pre-season, 7 weeks) (Iturricastillo et al., 2022). Considering that one of the main objectives of the coach is to achieve maximum performance at the most important moments of the season (competition period), it would be interesting to know how internal load could be related to changes in physical condition to determine the optimal amount of physiological load needed to enhance performance.

In this line of research, the main objectives of the study were: 1) to describe the differentiated perceived training and match load (dRPE-L) of WB players during the whole season; 2) to analyze the evolution of the players’ physical condition throughout the season and 3) to analyze the association between the dRPE-L and the change in the players physical condition during the season.

## 2 Material and methods

### 2.1 Participants

Nineteen Spanish Second Division WB players (16 men and 3 women; 27 ± 10 years; four to nine training h/wk) with at least 2 years’ training experience in WB participated in this study. The participants were classified according to the Classification Committee of the IWBF (category 1, n = 4; category 2, n = 2; category 2.5, n = 2; category 3, n = 4; category 3.5, n = 1; category 4, n = 4; category 4.5, n = 2) and had a valid license from the Spanish Federation of Sports for People with Physical Disabilities. All the values of the players who participated in the team training sessions and matches during the whole season were recorded and included for statistical analysis. This study was approved by the institutional research ethics committee, and all participants provided written informed consent as outlined in the Declaration of Helsinki (2013).

### 2.2 Procedures

**FIGURE 1:**
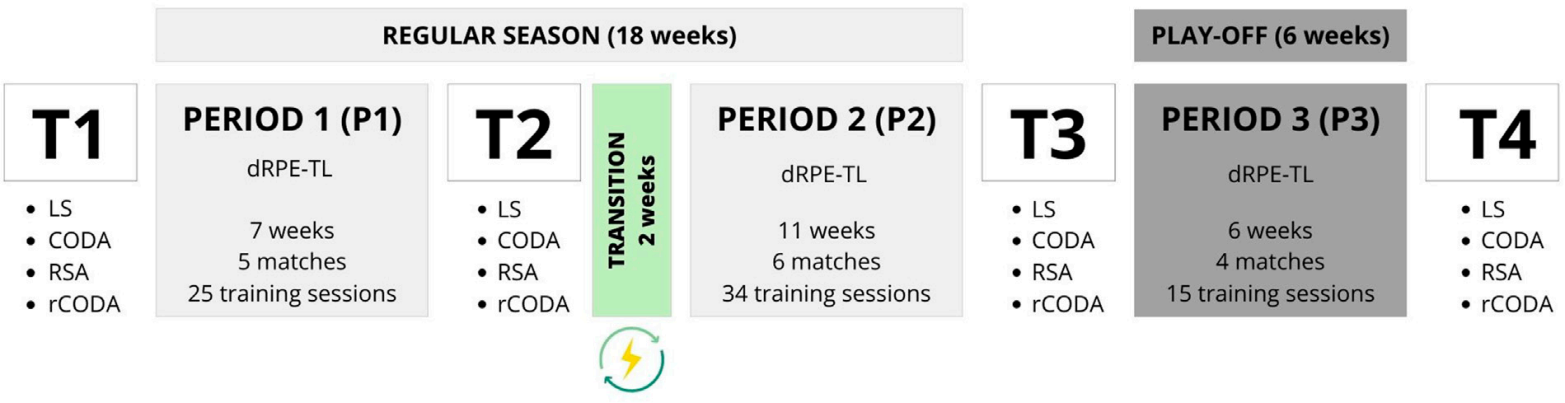
Description of the training and match program, measured training and match load and physical fitness assessment during the three periods of the entire season. T = test, dRPE-L = Differentiated perceived training and match load; LS = Linear Sprint Test; CODA = Change of Direction Ability; RSA = Repeated Sprint Ability; rCODA = Repeated Change of Direction Ability.

The data were collected in the WB season for 10 months (September to June) (Figure 1). Over the competitive season, participants’ dRPE-L values were recorded for all training and match sessions (15 matches and 74 training sessions were observed during the season). The season was divided into three periods: a first phase of the regular league (P1; 7 weeks, five matches, 25 training sessions), a second phase of the regular league (P2; 11 weeks, six matches, 34 training sessions) and a final play-off phase (P3; 6 weeks, four matches, 15 training sessions). Between P1 and P2 there was a transition period of 2 weeks (Christmas holiday) with a break from training and competition. At 4 different times during the season, the participants performed a standardized battery of tests on the training court to assess linear sprint, repeated sprint ability (RSA), change of direction ability (CODA) and repeated change of direction ability (rCODA): 1 week before the competitive period (T1), at the end of the first round of the regular season (T2), at the end of the regular season (T3) and at the end of the play-off phase for promotion to the first division (T4).

### 2.3 Training and match load quantification

The session-RPE method was used to calculate perceived training and match load as proposed by Foster et al. (2001) and previously used in WB players (Paulson et al., 2015; Iturricastillo et al., 2016). The players performed three sessions/week (Monday, Tuesday and Thursday), with an approximate duration of 150 min each session. The session started with 60 min of shooting and individual technique, and the rest of the session was destined to tactical-collective tasks. Fifteen minutes after every training session and match, players were asked to rate the perceived level of exertion using the CR-10 RPE scale, separately for respiratory (RPEres) and arm muscular (RPEmus) effort (Iturricastillo et al., 2016). All the players were familiarized with this method since it had been used by the staff in previous seasons and during pre-season training and friendly matches. Then, to calculate training or match dRPE-L, the total number of minutes of each training or match session was multiplied independently by the RPE score (RPEres-L and the RPEmus-L) given by each player. The total weekly load was calculated as the sum of the training and match load for each week. Also, in each period, the average weekly load with RPEres (RPEresAVG-L) and RPEmus (RPEmusAVG-L), and the total accumulated load with RPEres (RPEresTOT-L) and RPEmus (RPEmusTOT-L) were calculated respectively.

### 2.4 Physical fitness assessment

In order to analyze the players’ physical condition, they performed the same test battery at four different times during the season (T1-T4). The physical tests were performed in two different sessions, separated by at least 48 h in the same week. In the first session, the players performed the Repeated Sprint Ability (RSA) and Repeated Modified Agility *t*-test (rMAT) test and in the second session 3-3-6, 505 and Illinois Agility tests. All tests were performed on the same indoor track and in the same time slot (between 17 and 20 p.m.), with the individual competition wheelchair. All participants were instructed to perform the tests at maximum intensity. The 48 h prior to the tests, players did not perform any demanding physical activity. Before each testing session, players performed a standardized warm-up consisting of 5 min of wheelchair propulsion at low intensity, dynamic stretching and two acceleration drills.

#### 2.4.1 Linear sprint test

Three to six linear sprint test: The players had to perform a 12 m sprint divided into three short sprints of 3 m, 3 m and 6 m in a straight line (de Witte et al., 2018; Narvaez et al., 2021). Participants placed their chairs 0.5 m from the starting mark and had to stop the wheelchair completely between each sprint (de Witte et al., 2018; Narvaez et al., 2021). Each player performed two repetitions with 2 min of recovery between each attempt. The time (s) taken to complete the 12 m was measured with 0.4 m high two time gates (Witty, MicrogateTM, Bolzano, Italy) placed at the starting point (initial mark) and finish line (12 m). The best result of the two repetitions was used for statistical analysis.

#### 2.4.2 Change of direction ability (CODA) tests

***505 test:*** The previously proposed protocol for WB players was used (Iturricastillo et al., 2019; Narvaez et al., 2021). After performing a 10 m launched start (without measuring time), the players had to perform 5 m at maximum speed and at the line marked on the ground perform a 180° change of direction and finish the test by propelling themselves the 5 m back through the time gates. Time measurement was performed using 0.4 m high one time gate (Witty, MicrogateTM, Bolzano, Italy) and started and ended when the participant crossed the line between the gates. Participants completed two repetitions. The turning side in the first repetition was freely chosen by each player and had to be maintained in the rest of the repetitions. The recovery time between repetitions was 2 min. The best result of the two repetitions was used for statistical analysis.

***Illinois Agility Test*** (***IAT***) ***:*** The players had to perform the protocol previously proposed for wheelchair athletes (Usma-Alvarez et al., 2010; Narvaez et al., 2021). Participants placed their chairs 0.5 m from the starting line and were required to perform a course that included straight-line and multidirectional sprints, as well as movements around cones. The time (s) taken to complete the entire course was measured using 0.4 m high two time gates (Witty, MicrogateTM Witty, Bolzano, Italy). Participants completed two repetitions with a recovery time of 2 min. The best result of the two repetitions was used for statistical analysis.

#### 2.4.3 Repeated sprint ability (RSA) test

*Repeated Sprint Ability test (RSA):* A protocol previously used in WB players was employed (Narvaez et al., 2021). Participants were positioned at 0.5 m from the starting line and performed 12 sprints of 5 m with 10 s active rest between each repetition returning to the starting line. The time (s) spent in each sprint was recorded using 0.4 m high four time gates (Witty, MicrogateTM, Bolzano, Italy) at the start line and at 1 m, 3 m and 5 m from the start line. In order to avoid a drop in speed at the end of each sprint, participants were instructed to continue a further 1–2 m after passing 5 m. For the total number of sprints, the best sprint (RSAbest), the average of the 12 sprints (RSAavg) and the sprint decrement index (RSASdec) were taken into consideration (Iturricastillo et al., 2019). RSASdec was determined using this equation (Narvaez et al., 2021): RSASdec = $\left(\frac{RSAtotal}{RSAbest\;X\;12}x100\right)-100$

#### 2.4.4 Repeated change of direction ability (rCODA) test

*Repeated Modified Agility T-test (rMAT):* Players performed the protocol previously used by other authors in WB (Iturricastillo et al., 2021; Narvaez et al., 2021). The participants placed their wheelchairs at 0.5 m from the 0.4 m high time gate (Witty, MicrogateTM, Bolzano, Italy) placed at the start/finish point. The players performed 12 repetitions in a T-shaped path, starting the next repetition every 30 s. The total time (s) spent in each repetition was recorded using one time gate. In the seventh repetition, the direction of execution was changed. For the total number of sprints, the best sprint (rMATbest), the average of the 12 sprints (rMATavg) and the sprint decrement index (rMATSdec) were taken into consideration (Iturricastillo et al., 2021). rMATSdec was determined using this equation (Narvaez et al., 2021): rMATSdec = $\left(\frac{rMATtotal}{rMATbest\;X\;12}x100\right)-100$

### 2.5 Statistical analysis

Standard statistical methods were used to calculate of the mean and standard deviation (SD). Data were screened for normality of distribution and homogeneity of variances using the Shapiro-Wilk normality test and Levene test. A repeated measures ANOVA with Bonferroni *post hoc* was used to analyze the differences among the three periods (P1, P2 and P3) in terms of RPEres-L and RPEmus-L, and the results of each of the tests at the four measuring times (T1, T2, T3 and T4). A paired samples *t*-test was performed to determine the differences between RPEres-L and RPEmus-L in each period. In each case, the mean differences were calculated as a percentage (*Δ*%) = [(B–A) x 100/A]. Also, the effect size (ES) (Cohen, 1988) was calculated. The scale for the interpretation of the ES was: <0.2, trivial; 0.2 to 0.5, small; 0.5 to 0.8, moderate; and >0.8, large. Correlations between the dRPE-L values in each period and the percentage change in physical condition test results were calculated using the Spearman product moment correlation r). The following scale of magnitudes was used to evaluate correlation coefficients: <0.1, trivial; = 0.1–0.3, small; <0.3–0.5, moderate; <0.5–0.7, large; <0.7–0.9, very large; and <0.9–1.0, almost perfect (Hopkins et al., 2009). Data analysis was performed using the Statistical Package for Social Sciences (SPSS^®^, version 25.0 for Mac, Chicago, IL, United States of America). The *p* < 0.05 criterion was used for establishing statistical significance.

## 3 Results

**FIGURE 2:**
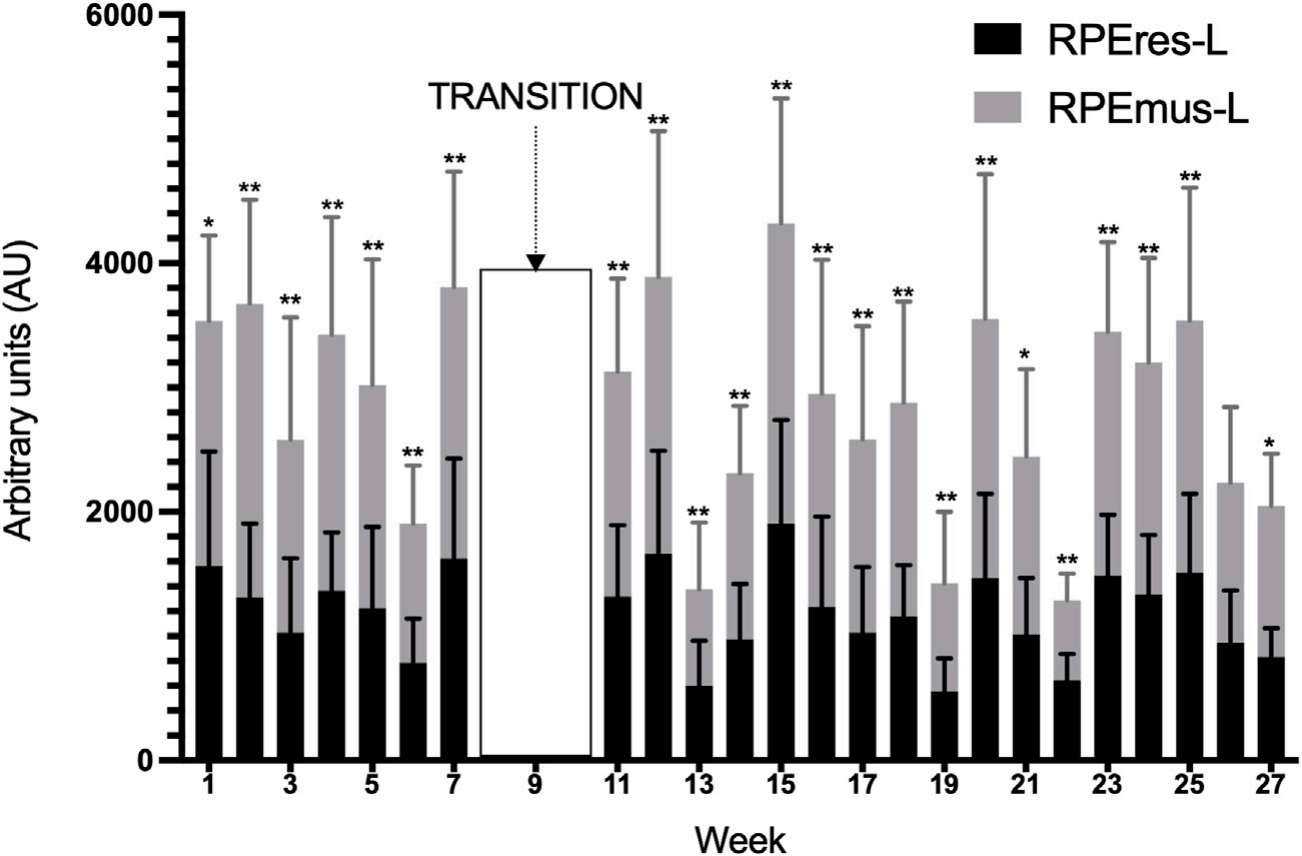
Description of the distribution of weekly training and match total load during the whole season. RPEres-L = perceived respiratory load; RPEmus-L = perceived muscular load. *p < 0.05, **p < 0.01 dif sig entre RPEresL y RPEmusL.

Team RPEresTOT-L and RPEmusTOT-L recorded during the whole season was 24,985.3 ± 11,526.1 AU and 33,265.3 ± 17,741.3 AU, respectively, while the RPEresAVG-L and dRPEmus-AVG-L was 1,050.5 ± 494.5 AU and 1,376.2 ± 741.9 AU. A significantly higher perceived load was observed in RPEmusTOT-L than RPEresTOT-L (*p* < 0.01; ∆% = 33.2; ES = 0.55, large). In this vein, players also reported a significantly higher perceived load in RPEmusAVG than RPEresAVG (*p* < 0.01; ∆% = 31.0; ES = 0.52, large). The dRPE-L values of each week across the whole WB season are shown in Figure 2. Except for week 26, higher values of RPEmus-L than RPEres-L were obtained in all weeks (*p* < 0.05, ∆% = 11.2–99.3; ES = 0.40–1.64, small–large).

Team total and weekly average dRPE-L for each period is presented in Table 1. For both RPEresTOT-L and RPEmusTOT-L, significantly higher values were obtained at P1 than at P3 (*p* < 0.05, ∆% > −23.5, ES > −0.41, moderate), at P2 than at P1 (*p* < 0.01, ∆% = 22.7, ES = 0.41 and *p* < 0.01, ∆% > 17.9, ES > 0.39, moderate, respectively) and at P2 than at P3 (*p* < 0.01, ∆% > −35.7, ES > −0,85, very large and *p* < 0.01, ∆% > −46.3, ES > −1,11, almost perfect, respectively). In the weekly average, although no differences were observed between the different periods in RPEresAVG-L, RPEmusAVG-L was higher in P1 with respect to P2 (*p* < 0.01, ∆% > −25.0 ES > −0.51, large) and P3 (*p* < 0.01, ∆% > −34.1, ES > −0.63, large) and higher in P2 than in P3 (*p* < 0.05, ∆% = −17.2, ES = −0.10, trivial). Significant differences were observed between RPEresTOT-L and RPEmusTOT-L, and between RPEresAVG-L and RPEmusAVG-L in P1 (*p* < 0.01, ∆% > 43.6, ES > 0.68, large) and P2 (*p* < 0.01, ∆% > 38.9, ES > 0.13, small).

**TABLE 1:** Differentiated perceived load (dRPE-L) results in relation to different periods of the season (P1-P3)*.*

	P1	P2	P3	P1 vs P2 *Δ* (ES)	P1 vs P3 *Δ* (ES)	P2 vs P3 *Δ* (ES)
RPEresTOT-L (AU)	8,459.1 ± 3,224.1	10,708.1 ± 5,049.3	7,160.8 ± 3,080.0	22.7 ± 0.5 (0.53)**	−23.5 ± 0.3 (−0.41)*	−35.7 ± 0.2 (−0.85)**
RPEmusTOT-L (AU)	12,145.3 ± 5,385.7	14,868.8 ± 8,166.5	7,694.0 ± 3,998.3	17.9 ± 0.4 (0.39)**	−43.5 ± 0.2 (−0.94)**	−46.3 ± 0.1 (−1.11)**
RPEres-L vs dRPEmus-L *Δ* (ES)	43.6 (0.68)**	38.9 (0.13)**	7.5 (0.51)	-	-	-
RPEresAVG-L (AU)	1,208.5 ± 460.6	973.5 ± 459.1	969.7 ± 513.3	−21.9 ± 0.3 (−0.51)	−10.7 ± 0.4 (−0.03)	2.3 ± 0.4 (0.45)
RPEmusAVG-L (AU)	1,735.1 ± 769.4	1,351.7 ± 742.4	1,282.8 ± 666.4	−25.0 ± 0.3 (−0.51)**	−34.1 ± 0.3 (−0.63)**	−17.2 ± 0.2 (−0.10)*
RPEres-L vs RPEmus-L *Δ* (ES)	43.6 (0.68)**	38.9 (0.13)**	7.5 (0.51)	-	-	-

*Note*. RPEres-L, perceived respiratory load; RPEmus-L, perceived muscular load; TOT-L, total load; AVG-L, weekly average load; P1 = first period of the season; P2 = second period of the season; P3 = third period of the season; *Δ* = mean difference in percentage; ES, effect size; *p < 0.05 and **p < 0.01 significant differences.

Table 2 shows the results obtained from the different physical tests recorded to analyze the evolution of players’ physical condition during the season. No significant differences were observed in any of the test variables recorded in different test sessions, except in RSASdec3m between T1 and T3 (*p* < 0.05, ∆% = −7.49, ES = −0.80, very large).

**TABLE 2:** The physical performance results in relation to different moments of the season (T1-T4).

	T1	T2	T3	T4	T1 vs T2 *Δ* (ES)	T1 vs T3 *Δ* (ES)	T1 vs T4 *Δ* (ES)	T2 vs T3 *Δ* (ES)	T2 vs T4 *Δ* (ES)	T3 vs T4 *Δ* (ES)
**Linear sprint**										
3-3-6 (s)	5.48 ± 1.00	5.98 ± 0.70	5.35 ± 0.42	5.43 ± 0.37	11.40 ± 0.06 (0.58)	6.77 ± 0.07 (0.17)	6.44 ± 0.06 (0.07)	−11.55 ± 0.08 (−1.09)	−4.17 ± 0.02 (−0,98)	0.59 ± 0.07 (0.20)
**Change of direction ability (CODA)**										
505 (s)	4.48 ± 0.59	5.33 ± 0.68	4.66 ± 0.33	4.56 ± 0.27	15.86 ± 0.11 (1.34)	6.17 ± 0.04 (0.38)	2.49 ± 0.06 (0.18)	−13.95 ± 0.08 (−1.25)	−9.81 ± 0.03 (−1.49)	0.11 ± 0.06 (0.33)
Illinois (s)	26.83 ± 2.44	27.46 ± 2.62	26.16 ± 1.82	26.22 ± 1.01	4.76 ± 0.08 (0.32)	0.12 ± 0.01 (0.25)	0.37 ± 0.02 (0.31)	−7.06 ± 0.08 (−0.56)	−4.17 ± 0.07 (−0.61)	0.70 ± 0.02 (0.41)
***Repeated sprint ability*** (***RSA***)										
*Total 12 sprints*										
RSAbest1m (s)	0.50 ± 0.05	0.48 ± 0.04	0.50 ± 0.05	0.47 ± 0.06	0.54 ± 0.11 (0.44)	5.41 ± 0.03 (0.00)	−8.44 ± 0.13 (−0.54)	4.15 ± 0.09 (0.44)	−8.27 ± 0.13 (−0.20)	−9.01 ± 0.12 (−0.54)
RSAbest3m (s)	1.25 ± 0.13	1.14 ± 0.29	1.22 ± 0.08	1.18 ± 0.09	−5.89 ± 0.26 (−0.49)	2.66 ± 0.04 (0.28)	−2.33 ± 0.06 (−0.63)	−0.22 ± 0.07 (−0.38)	0.23 ± 1.10 (0.19)	−3.98 ± 0.05 (−0.48)
RSAbest5m (s)	1.89 ± 0.20	1.79 ± 0.28	1.82 ± 0.15	1.76 ± 0.15	−2.85 ± 0.14 (−0.41)	1.28 ± 0.04 (0.40)	−3.80 ± 0.05 (−0.74)	10.06 ± 0.26 (0.13)	3.21 ± 0.19 (0.13)	−4.40 ± 0.04 (−0.4)
RSAavg1m (s)	0.53 ± 0.07	0.51 ± 0.04	0.54 ± 0.07	0.50 ± 0.05	1.29 ± 0.14 (0.35)	6.04 ± 0.07 (0.14)	−1.23 ± 0.08 (−0.49)	3.65 ± 0.12 (0.53)	−2.14 ± 0.09 (−0.22)	−7.18 ± 0.02 (−0.66)
RSAavg3m (s)	1.29 ± 0.15	1.26 ± 0.12	1.29 ± 0.14	1.23 ± 0.08	1.81 ± 0.09 (0.22)	6.29 ± 0.04 (0.00)	0.49 ± 0.05 (0.50)	−0.78 ± 0.06 (−0.23)	−2.12 ± 0.04 (−0.30)	−7.28 ± 0.02 (−0.53)
RSAavg5m (s)	1.96 ± 0.24	1.91 ± 0.17	1.92 ± 0.21	1.85 ± 0.13	1.44 ± 0.09 (0.24)	3.81 ± 0.04 (0.18)	−1.06 ± 0.04 (−0.57)	−2.72 ± 0.05 (−0.05)	−3.47 ± 0.04 (−0.40)	−5.69 ± 0.02 (−0.40)
RSASdec1m (%)	0.94 ± 0.27	0.84 ± 0.17	0.80 ± 0.33	0.93 ± 0.06	−14.68 ± 3.09 (−0.44)	−19.30 ± 4.74 (−0.47)	0.05 ± 3.80 (0.05)	−15.62 ± 0.42 (−0.15)	2.68 ± 0.05 (0.71)	−0.48 ± 0.08 (−0.55)
RSASdec3m (%)	1.03 ± 0.02	1.14 ± 0.88	0.95 ± 0.14	0.86 ± 0.16	−14.16 ± 0.92 (−0.17)	−7.49 ± 0.14 (−0.80)*	−14.68 ± 0.17 (−1.49)	−5.94 ± 0.14 (−0.30)	−21.17 ± 0.29 (−0.44)	−13.80 ± 0.16 (−0.60)
RSASdec5m (%)	1.03 ± 0.03	1.01 ± 0.27	1.04 ± 0.05	1.02 ± 0.03	−1.89 ± 0.28 (−0.11)	1.54 ± 0.05 (0.24)	−0.31 ± 0.04 (−0.33)	−8.03 ± 0.18 (−0.16)	−5.14 ± 0.17 (−0.05)	−4.21 ± 0.05 (−0.49)
***Repeated Modified Agility Test*** (***rMAT***)										
rMATbest (s)	9.48 ± 1.06	9.18 ± 0.31	9.22 ± 0.27	9.27 ± 0.63	0.75 ± 0.06 (0.39)	2.71 ± 0.06 (0.34)	0.45 ± 0.07 (0.24)	−0.47 ± 0.04 (−0.13)	−0.22 ± 0.06 (−0.18)	−3.28 ± 0.02 (−0.10)
rMATavg (s)	10.15 ± 1.06	9.54 ± 0.27	9.49 ± 0.20	9.80 ± 1.02	−1.84 ± 0.07 (−0.79)	−2.07 ± 0.06 (−0.87)	−2.43 ± 0.06 (−0.34)	−1.77 ± 0.02 (0.21)	−0.81 ± 0.06 (−0.35)	−2.74 ± 0.04 (−0.42)
rMATSdec (%)	7.48 ± 3.89	4.01 ± 0.62	2.53 ± 1.53	4.74 ± 5.99	−2.10 ± 0.07 (-0.90)	−67.95 ± 0.17 (−1.49)	−55.67 ± 0.27 (−0.55)	−38.27 ± 0.40 (−0.33)	−23.52 ± 0.38 (−0.14)	92.83 ± 1.52 (0.38)

*Note*. Avg = average; Sdec = sprint decrement index; T1 = testing session 1; T2 = testing session 2; T3 = testing session 3; T4 = testing session 4; *Δ* = the delta value; ES, effect size; *p < 0.05 significant differences between T1 and T4.

A significant association between total and average dRPE-L and physical condition delta value was found only in RSA during all periods without a clear pattern. A significant association was observed between the RPEresTOT-L of the whole season with the change in RSAbest5m between T1 vs T4 (r = 0.90, *p* < 0.05). At P1, a significant association was observed between total and mean RPEres-L with RSASdec1m, RSASdec3m and RSASdec5m (r > 0.67, *p* < 0.05). In P2, on the other hand, RPEres-L did not correlate with any variable while average and total RPEmus-L was related to RSAavg3m and RSAavg5m (r = 0.90, *p* < 0.05). Finally, in the latter part of the season (P3), RPEres-L and RPEmus-L did not correlate with any change in physical condition.

## 4 Discussion

The objectives of this study were to describe differentiated perceived training and match load (dRPE-L) of WB players during the whole season, to analyze the evolution of players’ physical condition changes during a full season and to analyze the association between dRPE-L and changes in physical condition during the season. Considering the scant evidence of longitudinal studies analyzing the effects of internal load on physical condition in WB, the main objectives of this study were to describe dRPE-L and physical condition changes during a WB full season and to analyze the association between dRPE-L and changes in physical condition during the season. The main findings were: a) a significantly higher dRPE-L was observed in P1 than P2 and P3, as well as in P2 than in P3 and the muscular perceived load being generally significantly higher than the respiratory perceived load in all periods; b) there were no significant changes in relation to physical condition except for the RSASdec3m; and c) significant associations were observed between RPEmus-L with RSAavg3m and RSAavg5m. To the best of the authors’ knowledge, this is the first study reporting relationships among the perceived load and physical condition changes in WB players for a whole competition season.

The dRPE-L is a practical tool to assess the internal load of training and matches (Foster et al., 2001; Los Arcos et al., 2015). Regarding changes in internal load among different periods of a competitive season in WB players using the dRPE-L method, a significant difference in RPEresTOT-L and RPEmusTOT-L was observed during the different periods, with P2 being the period with the highest total perceived load. However, this increased load is due to a higher number of training sessions and matches during this period. In this regard, contrary to the total load, players obtained a significantly higher RPEmusAVG-L in P1 compared to the other periods. These results are consistent with what has been observed in other team sport modalities. For example, Sansone et al. (2021) observed higher RPE values in the first part of the season in able bodied basketball players. Similarly, Los Arcos et al. (2015) observed a higher load in professional football players during the pre-season compared to the first part of the season. It appears that, as with other sports, the perceived weekly muscle load may be the highest of the entire season in the early part of the season. This difference could be due to the higher number of sessions (training and matches) in relation to the number of weeks in P1 compared to P2 and P3 (3.57, 3.09 and 2.05 sessions/week in P1, P2 and P3, respectively). For this reason, it could be interesting to carry out specific muscle strength training during the pre-season for WB players in order to increase the strength levels of the players and prepare them to tolerate future loads. Moreover, in terms of the differences between RPEmus-L and RPEres-L, a higher RPEmusTOT-L and RPEmusAVG-L compared to the RPEresTOT and RPEresAVG were observed for all periods. These results are consistent with another study carried out during specific training sessions with national first division WB players where they also observed higher values of RPEmus-L than RPEres-L during the pre-season (Iturricastillo et al., 2017). Once again, this idea is in line with the high muscular demands of WB training and matches, possibly due to the high muscular strength demands of propulsion and handling of the wheelchair during specific game actions (Gómez et al., 2014; Iturricastillo et al., 2015). It could be interesting to analyze the RPEmus and RPEres in order to analyze how the training and match load affects the peripheral-local and central-chest level in each player (Azcárate et al., 2020) and carry out preventive work for the upper-body muscles in order to avoid overload and/or injuries in players with a higher RPEmus.

The 3-3-6 linear sprint Test (de Witte et al., 2018; Narvaez et al., 2021), 505 Test (Iturricastillo et al., 2019; Narvaez et al., 2021) and Illinois Test (Usma-Alvarez et al., 2010; Iturricastillo et al., 2019), are some of the tests that traditionally have been used with WB players in order to assess physical condition. However, considering that WB is a sport characterized by high intensity intermittent efforts (Coutts, 1992; Seron et al., 2019), in addition to these simple tests, it could be interesting to use different tests that assess the ability to repeat maximum efforts in a straight line (RSA) (Iturricastillo et al., 2019; Narvaez et al., 2021) and changing direction (rCODA) (Iturricastillo et al., 2021; Narvaez et al., 2021). Although significant differences were observed in the perceived load between the different periods, in the physical condition, an anecdotical significant change was observed in the RSASdec1m variable (T1 vs T3). It is difficult to compare these results with other studies, as there has been no research using these tests longitudinally in WB. However, Iturricastillo et al. (2015) conducted a battery of tests at the beginning of the season (pre-test) and repeated it at the end of the season (post-test), without observing changes in the physical condition of first division WB players. In the same vein, in a study by Ayan et al. (2014), no significant differences were observed in different physical condition tests such as sprinting ability, CODA and endurance in first division WB players. Since in sports like WB the most important moment of the year often comes at the end of the season (i.e., play-offs), it could be interesting to try to progressively improve the physical condition of the players as the latter part of the season draws to a close. Possibly the reason for not having observed changes in the physical condition of the players throughout the season is that the team staff had carried out a training periodization focused on ensuring an adequate and stable physical condition of the players throughout the season. In this line, another reason could be that the tests may be conditioned by various physical and technical qualities. Perhaps the coaches intended to modulate the variables that affect the training internal load (volume, intensity, number of sessions/week and recovery) in order to have the players in an optimal state of physical condition for the whole season.

Quantifying and modelling the training load in team sports such as WB is an important aspect in order to know the appropriate training stimulus for each player and to avoid the negative consequences of an excessive training load (i.e., risk of injury and/or non-functional overreaching) (Ferioli et al., 2018). While significant changes in players’ perceived load were observed across periods (dRPETOT-L and RPEmusAVG-L), only an anecdotal significant association between perceived load and delta value in players’ physical condition was observed (in a single RSA variable). Despite the fact that in other team sports such as able-bodied basketball (Ferioli et al., 2018; Sansone et al., 2021) or in soccer (Los Arcos et al., 2015; Djaoui et al., 2017) the possible association between perceived load and physical condition has been analyzed, there are no studies to date to examine this aspect in WB players during a whole season. In WB players, Iturricastillo et al. (2022) evaluated the association between perceived load and changes in physical fitness over a shorter period (pre-season, 7 weeks). These authors, in the same line as this study, observed anecdotal associations between a few variables related to physical condition (sprint and lactate post in Yo-Yo IR1 test) and RPEmus. In studies carried out in different team sports (Los Arcos et al., 2015; Ferioli et al., 2018; Sansone et al., 2021), a negative association between internal load and changes in physical condition is observed. In this respect, Ferioli et al. (2018) observed a negative association between perceived load and neuromuscular response (measured by a jump test and CODA) of professional and semi-professional able bodied basketball players during a pre-season. Over a longer period, similar to this study (8 months), Sansone et al. (2021) observed the effects of the perceived training load on the performance of players during matches (through game statistics). In the same vein, these authors observed a negative association between perceived load and the direct performance obtained by players during matches. However, despite a negative association between perceived load and physical fitness parameters, contrary to other studies (Djaoui et al., 2017; Ferioli et al., 2018), the results of this study showed little significant change in physical condition over the course of the season. Despite a negative association between perceived load and physical fitness parameters, contrary to other studies (Djaoui et al., 2017; Ferioli et al., 2018), the results of this study showed little significant change in physical condition over the course of the season. Similarly, as observed in other studies (Djaoui et al., 2017; Ferioli et al., 2018; Sansone et al., 2021), the level of the participants or the playing position could be determining factors in the fitness response to the internal training and match load. Therefore, it might be interesting in future studies to analyze the effects of perceived load on changes in physical condition, taking into account the level, position and disability (or functional category) of the players, with the aim of determining the optimal load and the inclusion of specific training interventions to produce improvements in physical condition throughout the season; as long as they do not have a negative effect on competitive performance and the availability of players for matches.

Some limitations of this study must be taken into consideration. The results of this study should be taken with caution when extrapolating them to another team, as each player, depending on gender, type of disability or performance level, may behave differently. In this sense, the level of the team opponent could affect the internal load recorded during the matches. Therefore, it could be interesting to analyze this issue in future research. Although the RPE is a useful tool for quantifying training and match load, it is affected by personality, player characteristics, environmental and cognitive factors (Haddad et al., 2017). Consideration of these factors is important when interpreting the results, as well as familiarizing players with its use. Another limitation is that in this study no test was performed to evaluate the aerobic capacity of the players, being an important capacity in WB performance (Paulson and Goosey-Tolfrey, 2017).

## 5 Conclusion

The results of this study show significant changes in the perceived load (RPEmus-L) between the different periods throughout the season. In terms of physical condition, significant differences were only observed in some RSA variables at different times during the season. Despite the fluctuation in perceived load, significant associations were observed anecdotally between perceived load and some RSA variables. In future studies it could be interesting to analyze this association considering the characteristics of the WB players (level, position and type of disability).

## Data Availability

The original contributions presented in the study are included in the article/Supplementary Material, further inquiries can be directed to the corresponding author.
